# Cognitive abilities screening instrument-short form, mini-mental state examination and functional activities questionnaire in the illiterate elderly

**DOI:** 10.1590/S1980-57642013DN74000009

**Published:** 2013

**Authors:** Gabriela Pravatta Rezende, Juliana Cecato, José Eduardo Martinelli

**Affiliations:** 1 Graduanda em Medicina, na Faculdade de Medicina de Jundiaí (FMJ). Participante da Liga Acadêmica de Cardiologia da FMJ, Liga Acadêmica de Neurologia da FMJ e Primeira Secretária da Liga de Geriatria e Gerontologia da FMJ, Jundiaí SP, Brazil.; 2 Mestrado em Ciências da Saúde pela Faculdade de Medicina de Jundiaí (2013), possui graduação em Biologia pela Universidade São Francisco (2004) e Graduação em Psicologia pela Faculdade Anhanguera de Jundiaí (2012)., Jundiaí SP, Brazil.; 3 Doutorado em Educação pela Universidade Estadual de Campinas, Brasil (2008). Professor Colaborador da Faculdade de Medicina de Jundiaí, Brasil. Médico Responsável pela Disciplina de Geriatria e Gerontologia da FMJ e pelo Instituto de Geriatria e Gerontologia Comendador Hermenegildo Martinelli, Jundiaí SP, Brazil

**Keywords:** CASI-S, MMSE, PFAQ, aging, cognition

## Abstract

Dementia prevalence is increasing in developing countries due to population aging. Brief tests for assessing cognition and activities of daily living are very useful for the diagnosis of dementia by the clinician. Low education, particularly illiteracy, is a hindrance to the diagnosis of dementia in several regions of the world.

**OBJECTIVES:**

To compare the Brazilian version of the Cognitive Abilities Screening Instrument-Short Form (CASI-S) with the Mini-Mental State Examination (MMSE) and Pfeffer Functional Activities Questionnaire (PFAQ) for the diagnosis of dementia in illiterate elderly.

**METHODS:**

A cross-sectional study with illiterate elderly of both genders seen at the outpatient clinics of the Institute of Gerontology and Geriatrics Jundiaí, São Paulo state was performed. Spearman's correlation coefficient was used to correlate CASI-S, MMSE and PFAQ scores.

**RESULTS:**

The sample comprised 29 elderly over 57 years old whose mean scores on the CASI-S (scores ranging from 3 to 23) and the MMSE (scores ranging from 2 to 23) were 11.69 and 12.83, respectively. There was a strong significant correlation between the CASI-S and MMSE (r=0.75, p<0.001) and a moderate correlation coefficient that was significant and negative between the PFAQ and CASI-S (r= –0.53 p=0.003),similar to that between the MMSE and PFAQ (r= –0.41 p=0.025).

**CONCLUSION:**

The Brazilian version of the CASI-S demonstrates ease of application and correction in the illiterate elderly, and warrants further studies regarding its applicability for the diagnosis of dementia in populations with a heterogeneous educational background.

## Abstract (pt)

A prevalência de demência está aumentando nos países em desenvolvimento, devido ao envelhecimento da população. Testes breves para a avaliação da cognição e atividades de vida diária são muito úteis para o diagnóstico de demência pelo médico. Baixa escolaridade, especialmente analfabetismo, é um obstáculo para o diagnóstico de demência em diversas regiões do mundo.

**OBJETIVOS:**

Comparar a versão brasileira do teste Casi-S com o Mini Exame do Estado Mental (MMSE) e Questionário de Atividades Funcionais de Pfeffer (QAFP) para o diagnóstico de demência de analfabetos idosos.

**MÉTODOS:**

Estudo de corte transversal, com idosos de ambos os sexos, analfabetos, atendidos no Instituto de Geriatria e Gerontologia do município de Jundiaí, São Paulo. Foi utilizado o coeficiente de correlação de Spearman.

**RESULTADOS:**

A amostra foi composta por 29 idosos analfabetos, com médias de escore no Casi-S e no MEEM de 11,69 (variando de 3 a 23 pontos) e 12,83 (variando entre 2 a 23 pontos), respectivamente. Observou-se uma correlação forte e significativa entre o Casi-S e o MEEM (r=0,75; p<0,001) e um coeficiente de correlação moderado, significativo e negativo entre o Casi-S e o QAFP (r= –0,53; p=0,003), o que foi semelhante ao observado entre o MEEM e o QAFP (r= –0.41 p=0.025). Nota-se, portanto, uma correlação importante entre o MEEM e o Casi-S e valores moderados e significativos entre o desempenho cognitivo e o comprometimento nas atividades de vida diária.

**CONCLUSÃO:**

Observa-se que o Casi-S é um teste de fácil aplicação e correção, e que merece mais estudos sobre sua aplicabilidade no diagnóstico de demência em populações com heterogeneidade educacional.

## INTRODUCTION

The process of aging is currently a universal reality. Although observed initially in developed countries, today this phenomenon substantially affects developing nations such as Brazil.^1^ Increased life expectancy is undoubtedly clear evidence of improved population health, however, public health attention must focus on this age group of society, in order to assess and intervene in conditions to which it is subjected.^2^ Thus, some diseases more commonly seen in elderly, such as dementia, can be highlighted, requiring measures to assist these individuals. Therefore, it is critical to detect the diagnosis of dementia, for which cognitive tests are used to evaluate the cognitive abilities of the elderly. Epidemiological studies have shown that people over 65 with mental impairment have a higher chance of developing dementia, especially Alzheimer's disease (AD).^3^

The finding of cognitive and behavioral changes consistent with dementia is the basis for diagnosis. Cognitively preserved elderly do not differ in memory or on other cognitive domains, such as language, visuospatial abilities, praxis, gnosis, and executive functions. According to DSM IV (Diagnostic and Statistical Manual) criteria, those with dementia have cognitive defects, notably memory, affecting their daily life.^4^ AD is incorporated in this definition, and has a survival rate of, on average, eight years after the onset of symptoms.^5^ A study of Herrera et al.^6^ showed that AD represents over 50% of dementia cases. Duyckaerts et al.^7^ describe three stages of this disease, the first characterized primarily by memory changes in the episodic, semantic and language subsystems. The second step, in turn, is described by losses such as inability to solve problems, gnosis and executive function decline. Finally, the third stage is characterized by loss of autonomy and independence.

In this context, the CASI-S (Cognitive Abilities Screening Instrument - Short Form),^8^ along with the Mini-Mental State Examination (MMSE)^9^ and the Cambridge Cognitive Examination (CAMCOG),^10^ form the foundation for early diagnosis of AD, in particular during stage 1.7 The CASI-S includes: enrollment, temporal orientation, verbal fluency (four-legged animals in 30s) and recall (3 words). The maximum score is 33 points, and 23 points is the cut-off score for dementia in individuals younger than 70 years of age. In people aged over 70 years, the cut-off is now 20 points.^8^ It is a test used worldwide, especially in Brazil, and presents important features for describing cognitive deficits.

The studies by Teng et al.^11^ showed the great utility of the t CASI-S test, advocating its wider adaptation to different social and cultural groups and languages, in addition to its quick and easy application, without interference in its accuracy. It is noteworthy in this regard that in the current health reality, lengthy and tiresome neuropsychological tests should not be applied due to their relatively high cost and time involved.^11^ This study confirms these statements and addresses other important questions, such as comparison with the PFAQ, a widely used instrument but one that, as a survey of activities of daily living, has limitations compared to other cognitive tests, especially the CASI-S. Concurrently, we demonstrated the importance of integration between various tests for more reliable and specific AD diagnosis, highlighting the use of CASI-S as an aid in this regard, since it has high sensitivity and specificity and can be used reliably.

In 2005, a prospective study conducted in Honolulu with 3734 men aged between 71 and 93 years, showed the CASI-S was useful for reaching an early diagnosis of dementia, especially because of the decline of episodic memory, which had a low score 3 to 6 years before the clear onset of dementia in the subjects analyzed.^12^

In a recent literature review, the use of CASI in the investigation process in different countries is evident. Huang et al.^13^ analyzed the correlation between homocysteine in cortical perfusion and the risk for Alzheimer's disease in Taiwanese elderly.^13^ The CASI was used as a screening test for dementia in this population. In a Spanish population, the CASI was used to assess the cognition of patients with alcohol-related problems. This research indicated that the CASI proved an effective tool for investigating cognitive aspects in patients with drinking behavior.^14^ A Chinese version of the CASI was applied in a clinical practice to assess cognitive abilities and found a significant influence of education, suggesting the division of the instrument and establishing of cut-off points according to years of study.^15^ The instrument is also used as a cognitive screening in Latino adults with retinal microvascular abnormalities.^16^ In the study, the authors found a relationship between cognition and small blood vessels, in other words, low scores on the CASI-S were two times higher in patients with arteriolar narrowing and one time in patients with retinopathy signs.^16^

A study conducted by Damasceno et al.^17^ verified the relationship between primary reflexes and cognitive behavior in 30 elderly diagnosed with Alzheimer's disease. The CASI-S was one of the instruments used to assess cognitive aspects, demonstrating the condition was accompanied by decline in language, praxis, memory and orientation.^17^

Although there are international studies showing the importance of CASI-S in the evaluation of elderly patients with cognitive impairment, in Brazil there is only one study on this subject, calling for further research to be conducted in the country. This could broaden the use of CASI–S, since its utility is not restricted and can be applied in different contexts, with emphasis on basic health care, where this test could prevent a worsening of dementia, given the fact that it can provide early detection of decline in cognitive ability.

The objective of the present study was to analyze the efficiency of CASI-S in diagnostic investigations for Alzheimer's disease among illiterate elderly and to correlate this with other instruments applied in Brazil.

## METHODS

A cross-sectional study was conducted in illiterate elderly. This was a brief and descriptive study of cognitive performance based on screening tests validated for Brazil in a sample of patients with dementia in the Geriatric and Gerontology Outpatient Clinic of Jundiai Medical School. Elderly with schooling greater than one year and who could read and write were excluded. Upon first contact, patients underwent detailed clinical history-taking and were referred for neuropsychometric testing, which entailed application of the Mini-Mental State Examination (MMSE),^9^ CASI-S (Cognitive Abilities Screening Instrument - Short Form),^8^ Cambridge Cognitive Examination assessment (CAMCOG),^10^ the Geriatric Depression Scale (GDS) with 15 abbreviated items^18^ and Pfeffer Functional Activities Questionnaire (PFAQ).^19^

The diagnosis of dementia was established by DSM-IV^4^ and the criteria for Alzheimer's Disease, National Institute on Aging Alzheimer's Association^20^ were used. The PFAQ was conducted with the family member accompanying the patient in care and who also had daily contact, in order to collect as much information about performance in activities of daily living. The exclusion criteria were: patients aged younger than 56 years, history of stroke, Parkinson's disease, motor or visual impairment preventing patient from performing the cognitive tests, with depressive symptoms (GDS >8 points) and participants who did not agree to participate. The consent form was approved by the Research Ethics Committee (CEP) under process 54/11.

All data were analyzed using the SPSS (version 15.0) software package, which included conducting of descriptive analyzes of age, gender, and scores on the MMSE and CASI-S. The Kolmogorov-Smirnov test was also applied in order to verify the distribution of the sample, which indicated a parametric distribution. The Pearson correlation coefficient was established in order to compare the scores of CASI-S with variables of the MMSE and PFAQ.

## RESULTS

The sample comprised 29 elderly aged over 57 years of both sexes, where the majority of participants were female (82.8%) with the participation of 24 women. Mean age for the sample was 79.59 years (minimum=57, maximum=90, standard deviation [SD]=7.21) (Table 1). Regarding the scores for cognitive tests, the CASI-S had an average score of 11.69 (minimum=3, maximum=23, SD=5.23) while a mean of 12.83 was attained on the MMSE (minimum=2 , maximum=23, SD=4.54).

**Table 1:** Demographic data of sample regarding gender and age showing higher mean age and percentage among female subjects.

	%	Age (mean)	max	min	SD
Female	82.8	79.59 years	90	57	7.21
Male	17.2	79.36 years	89	62	6.37

Min: Minimum; Max: maximum; SD: standard deviation.

With respect to the tests applied, the CASI-S and MMSE, the Kolmogorov-Smirnov (KS) statistic was calculated to identify the sample distribution. The results indicated a normal distribution for the tests, as shown in Table 2. It can be inferred from the low number of participants that the results of the KS analysis followed a parametric distribution.

**Table 2:** Descriptive data of sample between CASI-S and MMSE by analyses of Kolmogorov-Smirnov test identifying sample distribution.

Variable	Mean	min	max	SD	P
CASI-S	11.69	3	23	5.23	0.586
MMSE	12.83	2	23	4.54	0.618

Min: Minimum; Max: maximum; SD: standard deviation.

A correlation test between the CASI-S, MMSE questionnaire and Pfeffer's Functional Activities Questionnaire (PFAQ) was performed. Table 3 shows a strong and significant correlation coefficient between the CASI-S and MMSE (r=0.75, p=0.000) and a moderate correlation coefficient that was significant and negative, between the PFAQ and CASI-S (r= –0.53, p=0.003). The results show a moderate coefficient that was significant and negative, between the MMSE and the PFAQ (r= –0.41, p=0.025). A significant correlation is evident between the MMSE and CASI-S, and significant and moderate values were found between cognitive performance and impairment in activities of daily living.

**Table 3:** Analyzes of Spearman's correlation coefficient among CASI-S, MMSE and PFAQ showing significant correlations between CASI-S and the other instruments.

Test	Analysis	CASI-S	MMSE	PFAQ
CASI-S	r	-	0.75	-0.53
	p	-	0.000	0.003
MMSE	r	-	0.75	-0.41
	p	-	0.000	0.025
PFAQ	r	-0.53	-0.41	-
	p	0.003	0.025	-

r: Spearman's Correlation; p: χ^2^.

## DISCUSSION

As shown in the statistical analyzes, the CASI-S had a strong significant correlation with the MMSE, having been effective in the diagnosis of AD. Since 2005, when it was first validated,^8^ the CASI-S has been widely used because of its practicality in clinical practice in hospitals and clinics, especially in public institutions, where appointment times are short and demand is high.^8^The areas covered by this test resemble those of the MMSE, involving aspects of enrollment, temporal orientation , verbal fluency and recall. The MMSE, in turn, includes, besides the mentioned aspects, local subtraction of sevens serially, object naming, phrase repetition, commands, reading, writing and drawing of pentagons. Thus, it is evident the MMSE is more detailed than the CASI-S which explains, in part, its wide application in Brazil since its creation in 1975 by Folstein et al., given it allows more detailed observation of the cognitive ability of the interviewed patient by including important data not addressed in the CASI-S. Furthermore, the MMSE has a high sensitivity and high specificity.^21,22^

CASI-S, however, is regarded as having greater ease and speed of implementation, and is also graduated to allow greater coverage of difficulty levels and does not involve writing, reading, drawing and calculation,^8^ which facilitates its use in elderly illiterate, whose score may be low and not indicating cognitive decline on the MMSE, but a limitation in relation to writing and reading.^8^ In this context, it is worth highlighting the advantages of the CASI-S compared to the MMSE.

With the MMSE, the respondent is requested to copy two pentagons, which makes writing something mandatory to score in this regard. During orientation, the first version of the test asks the current season,^9^ however, many patients do not even know how many seasons there are in one year, given the fact that this information is strongly influenced by educational level. Finally, the MMSE tests by applying a calculation (subtraction of sevens serially), which is undoubtedly determined by the formal education of the elderly individual performing the test.^21^ In this regard, we note that the MMSE depends greatly on the educational level of the respondent, while the illiteracy rate in Brazil remains high, especially in elderly.^22^ Thus, the MMSE has questionable applicability in the country, given that the IBGE (Brazilian Institute for Geography and Statistics) survey of 2010 revealed 14 million illiterates.

The level of education also influences the results on the CASI-S,^8^ however, its application has no written test, nor questions regarding seasons and calculations. Moreover, it proves faster, shorter and clearer with respect to its application in hospitals and outpatient geriatric and gerontology clinics.

Another important factor involves the MMSE scoring. The scale measures have been studied for years in Brazil, where different authors make use of scores which differ substantially. Bertolucci et al.^23^ established cut-offs that varied depending on the level of education, being determined as 13 points for illiterates, 18 for low and medium levels of schooling and 26 for high schooling level. This establishment was based on a study of application of the MMSE in 530 individuals. The study by Almeida et al.^24^ conducted in 211 individuals, established other cut-off scores, also related to the transcript. Thus, the cut-off among elderly illiterates was set at 19/20 and among those with schooling the score was 23/24.

These two studies show that the MMSE is also controversial in terms of scoring, comparable with the CASI-S, which has a score just described and so far not widely questioned and 23 points for under 70 years, with a sensitivity of 76.7% and specificity of 86.5%, and 21/20, with a sensitivity of 71.4% and specificity of 97.1%.^8^ This fact is not necessarily an advantage, but it is undeniable that the absence of disagreement over scores facilitates application and diagnosing dementia with the CASI-S.

The correlation of CASI-S and MMSE and the first test shows that, although not as widely applied as the latter, it offers features relevant for the diagnosis of AD akin to the MMSE, with easy application and being less objectionable in many respects, as previously mentioned.

The Pfeffer Functional Activities Questionnaire (PFAQ), in turn, evaluates the impact of dementia on patient's daily living. Thus, unlike the CASI-S and MMSE, which are cognitive tests of individuals, the PFAQ examines how the framework of cognitive impairment, proven by the tests mentioned above, affects the daily lives of the elderly. The PFAQ is applied, in most cases, to the elderly individual and family caregivers, in an attempt to achieve greater accuracy of responses. This is a problem because many seniors, even those with deficits in cognition, live alone or have no companion that can portray the correct picture of change in their life, or are not present in the daily lives significantly, or did not know their situation prior to dementia, requisites necessary in order to be conclusive about the impact of the dementia on the daily life of the elderly patient compared with the non-dementia stage. However, even if the test were applied directly to the elderly, tests of functional activity are not able to provide a longitudinal analysis of the development of dementia, because they do not assess the onset of dementia, but rather the stage of the patient on the day when the interview is conducted. Tests in this category, therefore, have no practical utility in the diagnosis of dementia, because, according to the DSM-IV, dementia is diagnosed by a decline in cognitive ability compared to a previous parameter. Tests such as the PFAQ are unable to perform a comparison between the onset of the dementia and the clinical status of the patient recently and therefore, according to DSM-IV, the single use of the PFAQ is insufficient to reach a diagnosis of dementia. The CASI-S, however, because it is a cognitive test, in the same category as the CAMDEX and MMSE, and given its extensive neuropsychological evaluations, has greater sensitivity in differentiating between normal subjects and early stages of demencia^25^ framework and can thus assist in the analysis of the PFAQ clinical status of the patient with suspected dementia.

Another important point that favors the CASI-S over the PFAQ and the MMSE is that it was validated in Brazil, while the others were developed in other countries which differs in relation to culture and education. Thus, it can be argued that the Casi-S is more suitable for the Brazilian milieu, not needing adaptations like the other tests, and it was created to cover most of the national population and the larger issues surrounding society such as level of education, for example. Moreover, in Brazil, primary health care is of major importance, and the CASI-S can be applied in households and health facilities through training of professionals and staff more easily than tests designed abroad and which have to be adapted for local use, where such adaptation does not always fully correspond to the reality in Brazil.^26^

This study thus demonstrates that the CASI-S, although not a test as broadly applied as the CAMDEX, MMSE or PFAQ, appears to be effective as a screening test, and offers unquestionable advantages, such as ease of application, short completion time thereby minimizing exhaustion of the interviewer and interviewee. Worldwide and particularly in Brazil, many cases of dementia go undiagnosed, which is explained by the lack of knowledge of the population about medicine and the application of cognitive tests for the diagnosis of dementia. In Brazil, the lack of conditions considered optimal for healthcare, ever shorter appointment times and a lack of interest from many doctors, especially general practitioners, accordingly, contributes to this scenario.^25^ As the instrument was developed by Brazilians, CASI-S, it is adapted to the Brazilian reality, being more suited to conditions involving the nation in the cultural population and medical society in general.

Another important point to note refers to the validation of the CASI-S as an effective screening test. According to Aprahamian et al.^27^ a good screening test for dementia should cover important aspects such as: speed of its administration, good tolerance and acceptance among patients; easy interpretation; cultural independence, language and education, high specificity and sensitivity values, presence of correlations with other traditional testing, well validated in the literature, and good predictive value, among others. Thus, the CASI-S fulfills most of the requirements necessary for a good screening test used in the diagnosis of dementia and can be used with ease, adding value to analyzes already accepted and established and is therefore useful in the diagnosis of cognitive impairment and dementia.

Is important to note, however, that this study had limitations, since it was applied to a small number of subjects being followed at the Geriatric and Gerontology Outpatient clinic of Jundiai Medical School, some already receiving treatment.

It can be concluded that, although this research ideally requires a larger number of participants, the present study demonstrates validity in proving a relationship between the CASIS and other tests with statistical significance, and is underway for future research. By conducting important comparisons, this study reinforces the validity of the CASI-S as a good and reliable cognitive test. Thus, the expanded use of CASI-S should be considered by clinicians, geriatricians and professionals working with seniors, for its greater ease and speed of cognitive testing, as an aid in the diagnosis of dementia, when used together with methods and questionnaires previously established and proven in Brazil. However, it is crucial that more studies are carried out along these lines with the objective of analyzing the real application of the test in basic health clinics, and the effect that early CASIS application can have in the diagnosis and treatment of dementia. In this way, CASI-S can have expanded use with reliable and effective results.
